# Modulatory efficacy of bone metabolism and safety of denosumab versus zoledronic acid in the treatment of bone and joint osteoporosis in elderly men

**DOI:** 10.3389/fphar.2025.1666421

**Published:** 2025-10-23

**Authors:** Jing He, Yuhui Mao, Wanran Gong, Haitao Zhu, Peng Xiang, Shui Wang, Xiaofeng Dai

**Affiliations:** ^1^ Department of Orthopedics, Sheyang County People’s Hospital, Yancheng, China; ^2^ Shanghai Jiao Tong University School of Medicine, Shanghai, China; ^3^ Key Laboratory of Neuroregeneration of Jiangsu and Ministry of Education, Coinnovation Center of Neuroregeneration, Medical School, Nantong University, Nantong, China

**Keywords:** osteoporosis, elderly men, denosumab, zoledronic acid, bone metabolism

## Abstract

**Objective:**

This study aimed to compare the clinical efficacy and safety of denosumab versus zoledronic acid (ZA) in the treatment of osteoporosis in elderly male patients, with the goal of optimizing therapeutic strategies for this population.

**Methods:**

A retrospective analysis was conducted on 89 elderly male osteoporosis patients treated at Sheyang County People’s Hospital from March 2023 to March 2024. Patients were allocated to two treatment arms based on the treatment regimen they received: the denosumab group (n = 49) and the ZA group (n = 40). Adverse drug reactions (e.g., myalgia, flu-like symptoms, and back pain) were recorded, and changes in bone metabolism markers and bone mineral density (BMD) at the lumbar spine (L1-L4), femoral neck, and total hip were evaluated after 1 year of treatment.

**Results:**

After 1 year of treatment, BMD of lumbar spine significantly increased in both groups (P < 0.05), with the denosumab group showing a greater improvement (0.41 ± 0.68 vs. 0.14 ± 0.86, P = 0.037). In contrast, the ZA group demonstrated superior efficacy in BMD of femoral neck (P = 0.011) and total hip (P = 0.029). Adverse reactions occurred in 15 patients: 2 in the denosumab group (muscle pain) and 13 in the ZA group (10 flu-like symptoms, 2 muscle pain, and 1 back pain). The incidence of flu-like symptoms was significantly higher in the ZA group (32.5% vs. 0%, P < 0.001).

**Conclusion:**

Denosumab demonstrated superior efficacy in reducing fracture risk at the lumbar spine, while ZA showed greater protective effects at the hip. Although both agents significantly improved bone metabolic parameters, denosumab exhibited a more favorable safety profile in clinical application.

## 1 Introduction

Osteoporosis (OP) is a common chronic metabolic osteopathy associated with multiple factors, including menopause and aging (Ni et al., 2024; Zhang et al., 2020; Wu et al., 2022; Jiang et al., 2021). Its clinical features include reduced bone density, altered bone quality, and abnormalities in microstructure and biomechanics (Tao et al., 2022; Guo et al., 2022; Yang et al., 2024; Kong et al., 2023).

Given that the patient population has historically shown a significant female-to-male ratio, the core target population for research is postmenopausal women, and some of the developed drugs (such as raloxifene) are even restricted to use in women. Treatment options for elderly male patients are extremely limited, particularly due to the need for further clarification of BMD response differences across different anatomical sites and the lack of comprehensive safety data. However, with further research in recent years, in June 2020, the National Medical Products Administration approved denosumab for therapeutic interventions for osteoporosis in aged male population with significant fracture risk (SJ20200020). Osteoporosis is one of the most common skeletal diseases among elderly men (Gao et al., 2022; Zhang et al., 2023; Chen et al., 2024; Bandeira et al., 2022; Johnston and Dagar, 2020). Pharmacotherapy is indispensable for fracture prevention in osteoporotic geriatric males. Denosumab and zoledronic acid (ZA), as the two most commonly used therapeutic drugs at present, have attracted considerable attention in clinical application (Lin et al., 2024). Denosumab binds receptor activator of nuclear factor-κB ligand (RANKL) with high specificity, suppressing osteoclast development and viability to decrease bone resorption and elevate bone mineral density (Kawahara et al., 2021; Hanley et al., 2012). ZA is a potent bisphosphonate drug used in clinical practice. It suppresses osteoclast activity, lowers bone turnover, and consequently elevates bone density (Wang et al., 2020). In traditional treatment, ZA is widely used, and its effectiveness in improving bone density has been clinically validated to a certain extent (Sølling et al., 2021). However, ZA may cause some adverse reactions during use, such as flu-like symptoms, muscle pain, and back pain (Jin et al., 2024). In elderly men, whose physical functions are relatively weak, this may affect their tolerance to treatment. Denosumab, as a relatively new drug, has shown good efficacy in increasing bone density (McConnell et al., 2022). However, the long-term efficacy of denosumab and its effects on different skeletal sites in elderly males require further research and discussion. Efficacy comparisons between denosumab and ZA for geriatric male osteoporosis lack consensus. Some studies suggest that denosumab is superior to ZA in improving spinal BMD (McConnell et al., 2022); however, other studies indicate that when considering drug safety, long-term efficacy, and effects on the entire skeleton, it is difficult to simply compare the advantages and disadvantages of them (Yassin et al., 2020). Therefore, how to precisely select the most appropriate treatment drug based on the individual differences of elderly male osteoporosis patients has become an important challenge for clinicians.

## 2 Methods

### 2.1 General data

This study included 89 elderly male patients with osteoporosis who received treatment at Sheyang County People’s Hospital from March 2023 to March 2024. A total of 320 patients preliminarily diagnosed with osteoporosis in elderly males who received treatment at our hospital between March 2023 and March 2024 were initially screened. After rigorous review of medical records, 231 patients were excluded due to incomplete data, severe complications, or loss to follow-up. Ultimately, 89 patients were included in the final analysis. Patients were assigned to either denosumab group (n = 49; age 73.6 ± 5.9 years, range 65–84) or ZA group (n = 40; age 72.5 ± 5.8 years, range 65–82) based on the treatment regimen they received. Baseline characteristics showed no significant intergroup disparities (P > 0.05; Table 1). The Institutional Review Board of Sheyang County People’s Hospital granted ethical approval (Approval Number: YCKY2430).

**TABLE 1:** Comparison of baseline characteristics between two groups.

Parameter	Denosumab group (n = 49)	ZA group (n = 40)	t-value	P-value
Age (years, $\bar{x}$ ±s)	73.6 ± 5.9	72.5 ± 5.8	0883	0.38
BMI/(kg/m^2^, $\bar{x}$ ±s)	21.45 ± 3.95	21.32 ± 3.23	0.19	0.85
Serum Calcium/(mmol·L^-1^, $\bar{x}$ ±s)	2.26 ± 0.10	2.28 ± 0.13	0.88	0.38
Serum Phosphate/(mmol·L^-1^, $\bar{x}$ ±s)	1.23 ± 0.16	1.21 ± 0.13	0.67	0.51
^25-(OH)D/^(ng·mL^-1^, $\bar{x}$ ±s)	25.24 ± 8.55	25.45 ± 8.82	−0.12	0.91
PTH/(ng·mL^-1^, $\bar{x}$ ±s)	44.23 ± 23.73	44.43 ± 16.42	−0.05	0.96
ALP/(U·L^-1^, $\bar{x}$ ±s)	73.35 ± 21.43	74.92 ± 23.40	−0.35	0.73
Lumbar Spine (L1-L4) T-score( $\bar{x}$ ±s)	−2.02 ± 0.41	−2.07 ± 0.33	0.65	0.52
Femoral Neck T-score( $\bar{x}$ ±s)	−2.11 ± 0.32	−2.21 ± 0.29	1.57	0.12
Total Hip T-score( $\bar{x}$ ±s)	−2.08 ± 0.26	−2.18 ± 0.27	1.81	0.08

Age refers to the age at the time of initial data collection.

### 2.2 Inclusion and exclusion criteria

**FIGURE 1:**
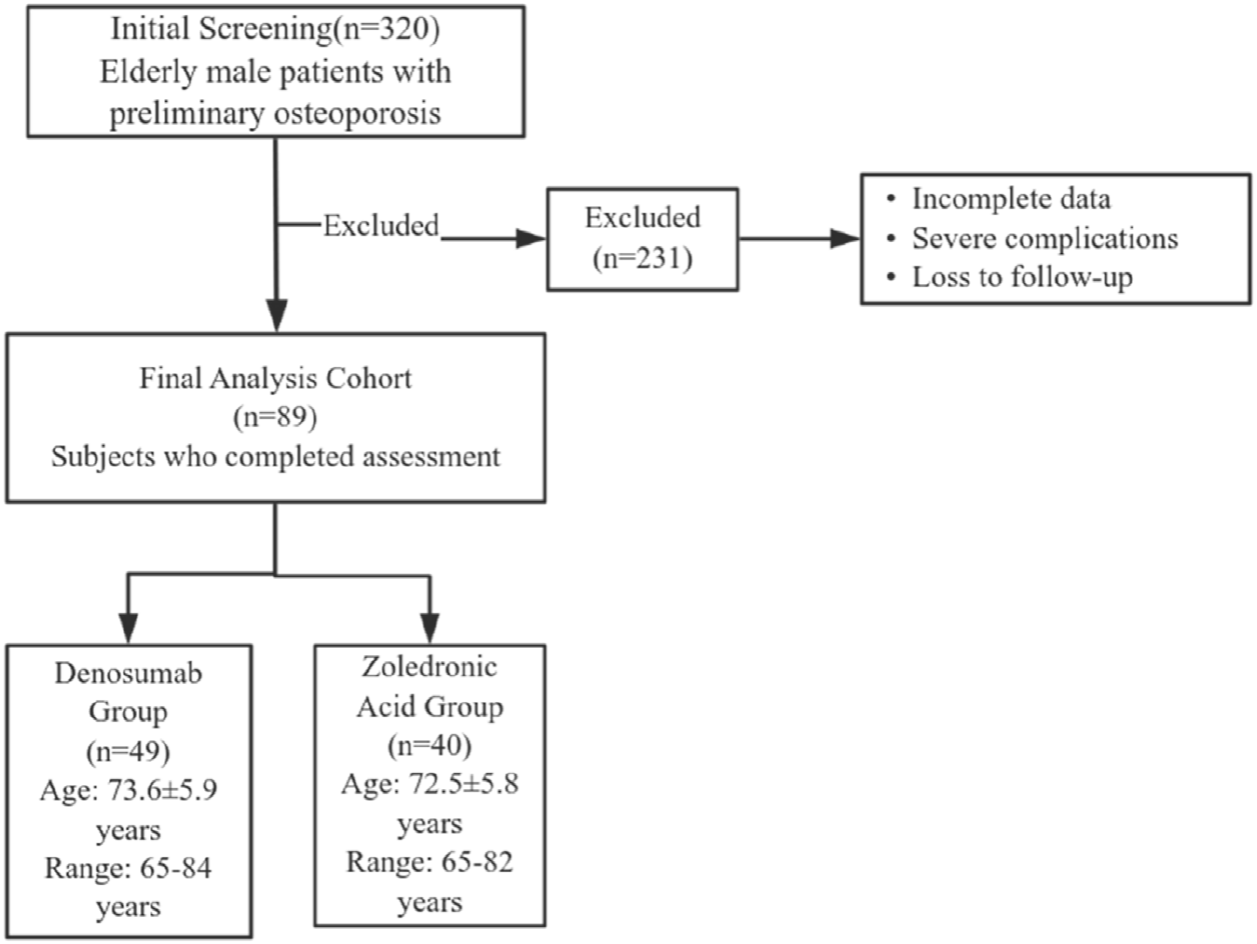
Patient inclusion and exclusion flow diagram.

The patient enrollment and exclusion process is illustrated in Figure 1. Inclusion criteria are: ① Patients meet the diagnostic criteria for OP as outlined in the “Diagnosis and Management of Osteoporosis (2015)” (Jeremiah et al., 2015); ② Age ≥65 years; ③ BMD assessment revealing osteoporosis-range T-score (<−2.5). Exclusion criteria are: ① Secondary osteoporosis (e.g., corticosteroid use, endocrine disorders, inflammatory processes, etc.); ② Failure to obtain informed consent; ③ Severe concomitant cardiovascular, pulmonary, hepatic, renal, or neurological conditions (e.g., heart failure, myocardial infarction, pulmonary edema, acute kidney injury, chronic kidney disease); ④ Presence of severe hematological disorders (e.g., leukemia, multiple myeloma, etc.); ⑤ Patients with metal implants in the hip or spine that may affect the accuracy of bone density measurements.

### 2.3 Treatment methods

All patients were treated by a designated physician. On the morning of the treatment day, a designated nurse drew fasting blood samples and administered medication to minimize procedural errors.

Denosumab group: Denosumab (60 mg) was administered via subcutaneous injection. Patients were observed for 30 min post-injection; if no adverse reactions occurred, they were discharged. Patients were instructed to return for follow-up if any adverse reactions developed within 1 week. A second subcutaneous injection of 60 mg was administered 6 months later.

ZA group: ZA (5 mg) was administered via intravenous infusion, mixed with 1,000 mL of 0.9% sodium chloride solution. Patients were observed for 24–48 h post-infusion. If no adverse reactions occurred, they were discharged. Patients were instructed to seek immediate medical attention if any adverse reactions occurred within 1 week.

During the treatment period, both groups of patients routinely took oral vitamin D calcium chewable tablets (each tablet contains 125 IU of vitamin D3 and 1.5 g of calcium carbonate, one tablet daily). Both groups of patients were treated for 1 year.

### 2.4 Evaluation criteria

Baseline assessment includes patient age, body mass index (BMI), and medical history. All subjects underwent dual-energy X-ray absorptiometry (DXA) scanning for lumbar (L1-L4), femoral neck, and total hip BMD at baseline and post-treatment year 1. Fasting morning specimens were obtained pre-dose and at 12-month follow-up for quantification of serum analytes: calcium, phosphate, 25-hydroxyvitamin D [25-(OH)D], parathyroid hormone (PTH), and bone-specific alkaline phosphatase (ALP). All participants were followed up via telephone or outpatient visits to record adverse reactions following subcutaneous injection of denosumab and intravenous infusion of ZA, including muscle pain, flu-like symptoms, back pain, etc., and timely targeted treatment was provided.

### 2.5 Statistical analysis

The data from this study were analyzed using SPSS 22.0 statistical analysis software. Categorical variables were compared intergroup with χ^2^ tests. Continuous variables were summarized as mean ± SD. To further assess differences, intergroup data were analyzed using the independent samples t-test, and intra-group changes were analyzed using the paired samples t-test. Statistical significance was set at P < 0.05 (two-tailed) for all tests.

## 3 Results

### 3.1 BMD results before and after treatment

The study cohort included 89 patients, with 49 in the denosumab group and 40 in the ZA group, all of whom were followed up for 1 year. After treatment, BMD at the lumbar spine (L1-L4) improved significantly in both the denosumab and ZA groups (P < 0.05); and total hip BMD in the denosumab group also showed a significant increase (P < 0.05). Comparisons of pre- and post-treatment changes between the two groups revealed that the increase in lumbar spine (L1-L4) BMD in the denosumab group was significantly greater than that in the ZA group (0.41 ± 0.68 vs. 0.14 ± 0.86) (P < 0.05), while the ZA group showed greater improvements in femoral neck BMD and total hip BMD compared to the denosumab group (P < 0.05), as shown in Table 2.

**TABLE 2:** Comparison of bone mineral density before and after 1-year treatment between two groups.

Group	Lumbar spine (L1-L4) T-score( $\bar{x}$ ±s)	Femoral neck T-score( $\bar{x}$ ±s)	Total hip T-score( $\bar{x}$ ±s)
Denosumab group (n = 49)			
Before treatment	−2.02 ± 0.41	−2.11 ± 0.32	−2.08 ± 0.26
1 year after treatment	−1.64 ± 0.61	−2.07 ± 0.29	−2.01 ± 0.27
*t-value*	5.782	0.442	1.912
*P-value*	<0.001	0.661	0.030
ZA group (n = 40)			
Before treatment	−2.07 ± 0.33	−2.21 ± 0.29	−2.18 ± 0.27
1 year after treatment	−1.82 ± 0.32	−2.16 ± 0.31	−2.15 ± 0.28
*t-value*	3.243	1.573	1.812
*P-value*	0.002	0.124	0.078
Change after treatment			
Before treatment	0.41 ± 0.68	0.03 ± 0.38	0.07 ± 0.39
1 year after treatment	0.14 ± 0.86	0.36 ± 0.92	0.03 ± 0.83
*t-value*	2.112	−2.642	−2.204
*P-value*	0.037	0.011	0.029

### 3.2 Pre- and post-treatment test results

Post-treatment bone metabolism markers exhibited differential responses: Both denosumab and ZA groups demonstrated significant ALP reduction (P < 0.05), while only denosumab significantly decreased 25-(OH)D levels (P < 0.05). No other markers showed statistically significant changes in either group (P > 0.05). Comparisons of pre- and post-treatment changes between the two groups showed no statistically significant differences in serum calcium, serum phosphorus, 25-(OH)D, PTH, or ALP (P > 0.05), as shown in Table 3.

**TABLE 3:** Comparison of bone metabolic markers before and after 1-year treatment between two groups.

Group	Serum Calcium/(mmol·L^-1^, $\bar{x}$ ±s)	Serum Phosphate/(mmol·L^-1^, $\bar{x}$ ±s)	^25-(OH)D/^(ng·mL^-1^, $\bar{x}$ ±s)	PTH/(ng·mL^-1^, $\bar{x}$ ±s)	ALP/(U·L^-1^, $\bar{x}$ ±s)
Denosumab group (n = 49)					
Before treatment	2.26 ± 0.10	1.23 ± 0.16	25.24 ± 8.55	44.23 ± 23.73	73.35 ± 21.43
1 year after treatment	2.30 ± 0.16	1.21 ± 0.17	29.14 ± 10.43	37.48 ± 15.38	64.42 ± 22.49
*t-value*	−1.428	0.588	−2.012	1.704	2.079
*P-value*	0.159	0.559	0.049	0.094	0.042
ZA group (n = 40)					
Before treatment	2.28 ± 0.13	1.21 ± 0.13	25.45 ± 8.82	44.43 ± 16.42	74.92 ± 23.40
1 year after treatment	2.23 ± 0.14	1.16 ± 0.17	27.62 ± 9.21	42.55 ± 16.82	65.05 ± 20.12
*t-value*	1.786	1.542	−1.175	0.532	2.188
*P-value*	0.081	0.131	0.247	0.598	0.034
Change after treatment					
*t-value*	1.972	1.392	0.112	1.441	0.159
*P-value*	0.052	0.168	0.911	0.153	0.874

### 3.3 Safety outcomes before and after treatment

Based on follow-up for adverse reactions after administration, two cases (4.1%) of adverse reactions were observed in the denosumab group, both of which were muscle pain; 13 cases (32.5%) of adverse reactions were observed in the ZA group, including 10 cases of influenza-like symptoms, two cases of muscle pain, and one case of back pain. Flu-like symptoms occurred more frequently in the ZA group (25%) than denosumab group (0%; P < 0.001). No intergroup differences emerged for back pain or myalgia (P > 0.05, Table 4).

**TABLE 4:** Comparison of adverse reactions before and after 1-year treatment between two groups.

Group	Back pain	Muscle pain	Flu-like symptoms
Denosumab Group (n = 49)	0	2	0
ZA group (n = 40)	1	2	10
*P-value*	0.45	1.00	<0.001

## 4 Discussion and conclusion

A comparison of the efficacy and safety of denosumab and ZA in elderly male patients with osteoporosis during knee arthroplasty revealed significant differences in the improvement of bone mineral density (BMD) at different sites between the two drugs. The results of this study showed that denosumab was significantly superior to ZA in improving lumbar spine BMD (P = 0.037). This finding is consistent with the conclusions of several recent clinical studies (Tsourdi et al., 2020), as it specifically inhibits the RANKL signaling pathway, blocking osteoclast differentiation and activation. Its effects encompass both cortical and trabecular bone, particularly demonstrating significant inhibitory effects on bone resorption in the highly metabolically active spinal column. The notable effect of ZA on femoral neck BMD (P = 0.011) may be related to its preference for targeting high bone turnover regions (such as the trabecular bone of the femoral neck) (Reid et al., 2018; Bolland et al., 2025), which reduces bone resorption by inhibiting osteoclast function.

It is worth noting that the incidence of influenza-like symptoms in the ZA group was as high as 25% (P < 0.001), which may be associated with the activation of γδ-T cells by bisphosphonates to release pro-inflammatory factors such as IL-6 (Campisi et al., 2022; Shiraki et al., 2021). In contrast, denosumab, which does not directly act on the immune system, has a significantly lower incidence of adverse reactions (4.1%) and is more suitable for elderly patients (Cummings et al., 2009). Besides, denosumab alone significantly reduced 25-(OH)D levels. It is speculated that these changes in 25-(OH)D may be related to feedback regulation triggered by alterations in calcium metabolism, and may also involve dilution effects or redistribution of vitamin D. Additionally, although no cases of severe hypocalcemia were reported in the denosumab group, serum calcium levels were significantly elevated compared to baseline (P = 0.048), suggesting the need for individualized adjustment of calcium supplementation, particularly in patients with impaired renal function.

The findings of this study partially align with the previous finding that denosumab demonstrates greater efficacy in improving spinal BMD (McConnell et al., 2022). However, it was also observed that ZA exhibited more pronounced therapeutic effects on lower limb bones. Nevertheless, Miller noted that the long-term safety profiles of both drugs (e.g., osteonecrosis of the jaw, atypical fractures) require further validation (Yassin et al., 2020). The limitations of this study include: ① the single-center retrospective design may lead to selection bias; ② the follow-up period was short (only 1 year), making it difficult to assess long-term efficacy and rare adverse reactions; ③ the sample size was small (n = 89), resulting in insufficient power for subgroup analysis; ④ ZA administration protocol differs from standard clinical practice, which may may limit the direct applicability of results; ⑤ no specific adverse reactions were recorded, nor were their severity or intervention measures documented. Future studies should include multicenter, large-sample, long-term research, particularly focusing on efficacy differences in patients with chronic kidney disease or diabetes (Chen and Sambrook, 2011), to clarify the drug’s applicability across different populations. Future adequately powered randomized controlled trials with longer follow-up period are needed to validate the comparative effectiveness and safety of denosumab versus zoledronic acid in elderly male patients. Additionally, our modified zoledronic acid administration protocol may limit generalizability to settings using standard protocols, warranting further studies with standardized dosing regimens.

From a clinical practice perspective, this study suggests that drugs should be selected individually based on the patient’s fracture risk location: patients at high risk of lumbar spine fractures should prioritize denosumab, while those at higher risk of hip or femoral neck fractures may consider ZA. Additionally, enhancing medication adherence management is crucial. A meta-analysis noted that denosumab must be strictly administered every 6 months to prevent bone density rebound (Chen et al., 2025; Burckhardt et al., 2021), while 10-year follow-up data for ZA showed its hip protection effect persists for 5 years post-discontinuation (Black et al., 2012). For elderly patients with poor tolerance, denosumab may be more advantageous due to fewer adverse reactions. Future research may explore combination therapy regimens (e.g., denosumab combined with teriparatide) to synergistically improve whole-body bone density (Sun et al., 2022), while also incorporating pharmacoeconomic assessments to optimize treatment costs.

In conclusion, our study found that denosumab led to superior bone mineral density (BMD) improvements in the lumbar spine, and ZA showed greater BMD improvements at the hip. These findings suggest a differential impact on fracture risk reduction based on anatomical site, with denosumab potentially offering more protection for lumbar spine fractures and ZA for hip fractures. Though both agents significantly improved bone metabolic parameters, denosumab exhibited a more favorable safety profile in the clinical application.

## Data Availability

The original contributions presented in the study are included in the article/supplementary material, further inquiries can be directed to the corresponding author.
